# Lung-diffusing capacity for carbon monoxide predicts early complications after cardiac surgery

**DOI:** 10.1007/s00595-019-1770-z

**Published:** 2019-01-31

**Authors:** Toshiyuki Kuwata, Ikuko Shibasaki, Koji Ogata, Hironaga Ogawa, Yusuke Takei, Masahiro Seki, Yuriko Kiriya, Hirotsugu Fukuda

**Affiliations:** Department of Cardiac and Vascular Surgery, Dokkyo Medical University, 880 Kitakobayashi, Mibu, Tochigi 321-0293 Japan

**Keywords:** Cardiac surgery, Diffusing capacity of lung for carbon monoxide, Complication

## Abstract

**Purpose:**

Preoperative pulmonary dysfunction has been associated with increased operative mortality and morbidity after cardiac surgery. This study aimed to determine whether values for the diffusing capacity of the lung for carbon monoxide (DL_CO_) could predict postoperative complications after cardiac surgery.

**Methods:**

This study included 408 consecutive patients who underwent cardiac surgery between June 2008 and December 2015. DL_CO_ was routinely determined in all patients. A reduced DL_CO_ was clinically defined as %DL_CO_ < 70%. %DL_CO_ was calculated as DL_CO_ divided by the predicted DL_CO_. The association between %DL_CO_ and in-hospital mortality was assessed, and independent predictors of complications were identified by a logistic regression analysis.

**Results:**

Among the 408 patients, 338 and 70 had %DL_CO_ values of ≥ 70% and < 70%, respectively. Complications were associated with in-hospital mortality (*P* < 0.001), but not %DL_CO_ (*P* = 0.275). A multivariate logistic regression analysis with propensity score matching identified reduced DL_CO_ as an independent predictor of complications (OR, 3.270; 95%CI, 1.356–7.882; *P* = 0.008).

**Conclusions:**

%DL_CO_ is a powerful predictor of postoperative complications. The preoperative DL_CO_ values might provide information that can be used to accurately predict the prognosis after cardiac surgery.

**Clinical trial registration number:**

UMIN000029985.

## Introduction

Preoperative pulmonary dysfunction in chronic obstructive pulmonary disease (COPD) has been considered to be associated with increased operative mortality and morbidity after cardiac surgery. A careful evaluation of the pulmonary function before and after cardiac surgery demonstrated a significant reduction in lung volume, diffusion capacity, and oxygenation at 2 weeks after surgery, with partial improvement after 4 months [1]. The preoperative identification of patients who are at greater risk of developing complications is important to prevent postoperative complications and obtain a good operative outcome.

The analysis of the diffusing capacity of the lung for carbon monoxide (DL_CO_) is a clinically useful pulmonary function test (PFT). Unlike other spirometric measurements, DL_CO_ is less influenced by patient effort [2]. DL_CO_ represents the ability of the lung to diffuse carbon monoxide across its membranes and assesses the transfer of gases from the alveoli to red blood cells. The diffusion of O_2_ depends on the following factors: the alveolar ventilation/capillary perfusion ratio, which establishes the partial pressure gradient of O_2_ between the alveoli and plasma; the physical characteristics of the alveolar–capillary interface; the capillary blood volume available for gas exchange; the hemoglobin (Hb) concentration; and the reaction rate between O_2_ and Hb [3, 4]. The diffusion characteristics of the lung are commonly assessed by tests of CO transfer. CO diffuses across the alveoli and binds to Hb with 240-fold greater affinity than O_2_ [3]. DL_CO_ depends on two resistances arranged in series according to the following equation:

1/DL_CO_ = 1/D_M_ + 1/*θ*_CO_*V*_C_ [3–5], where *D*_M_ is the alveolar–capillary membrane conductance, *θ*_CO_ is the rate of CO uptake by the whole blood combined with Hb measured in vitro, and *V*_C_ is the lung capillary blood volume [3–5]. A decline in DL_CO_ can occur as a result of destruction of alveolar structures, distal airway dysfunction, contraction of the pulmonary capillary volume due to ventilation, perfusion abnormalities, and Hb abnormalities.

DL_CO_ is an equally powerful predictor of postoperative complications in patients with and without COPD after lung resection. A previous study suggested that DL_CO_ should be routinely measured during preoperative evaluations, regardless of whether a patient’s spirometric values are abnormal [2].

Another study reported that reduced alveolar–capillary membrane conductance is associated with pulmonary congestion [6]. Thus, DL_CO_ may be influenced by pulmonary edema and fluid accumulation in the interstitial spaces before and after cardiac surgery. The present study aimed to determine whether DL_CO_ can serve as a predictor of complications arising after cardiac surgery.

## Patients and methods

### Patients

The study protocol was approved by the Institutional Review Board of the Dokkyo Medical University. Between June 2008 and December 2015, 2040 patients underwent cardiac surgery at Dokkyo Medical University Hospital. A total of 408 patients in whom preoperative DL_CO_ values were routinely collected within 1 week before scheduled cardiac surgery were included in this study. The attending physician for each patient made the decision to proceed with the PFT, which included measurement of DL_CO_, based on clinical indications. The exclusion criteria were any emergency or urgent operation, aortic surgery, beating heart surgery, and approaches other than median sternotomy. We reviewed the medical records of the patients, including the demographics, preoperative clinical data, PFT findings, hemodynamic data from cardiac catheterization, and operative and postoperative data.

### DL_CO_ measurement and %DL_CO_

We measured DL_CO_ in a single-breath-hold maneuver with the patient seated upright in a chair with their nostrils closed with a clip. The patients then breathed normally and exhaled to residual volume, and then, a carbon monoxide–helium mixture was forcefully inhaled to total lung capacity, and held for 10 s and then exhaled. The patients exhaled to wash out the estimated mechanical and anatomical dead space. Alveolar samples were then collected, and DL_CO_ was calculated from the total volume of the lung, breath-hold duration, and the initial and final alveolar concentrations of CO. The exhaled helium concentration was used to determine a single-breath estimate of the total lung capacity and the initial alveolar concentration of CO. The predicted DL_CO_ was determined from regression equations according to age, height, and sex (predicted DL_CO_ for men, 15.5 ×  body surface area (BSA) − 0.23 ×  age + 6.8; predicted DL_CO_ for women, 15.5 × BSA − 0.117 ×  age + 0.5) [7]. %DL_CO_ was calculated by dividing the actual DL_CO_ by the predicted DL_CO_.

### Surgical technique

A median sternotomy approach was applied under general anesthesia to all patients. Cardiopulmonary bypass (CPB) was established through the ascending aorta or by right atrial or bicaval cannulation. The myocardium was protected by antegrade and retrograde cardioplegia with intermittent cold-blood cardioplegia and reperfusion with warm-blood cardioplegia. A normothermic temperature was maintained during CPB. The patients were transferred to the intensive care unit immediately after the procedure with ventilator assistance and monitoring.

### Definitions of complications

Postoperative outcomes were defined according to the Society of Thoracic Surgeons National Database as follows. In-hospital death was defined as the death of a patient due to any cause during hospitalization in the institution, where they underwent cardiac surgery. Stroke was defined as a central neurologic deficit persisting for > 72 h. Wound infection was defined as infection involving subcutaneous tissue, muscle, bone, or the mediastinum, and requiring surgical intervention. Respiratory complications were also included. The incidence of postoperative respiratory complications was scored on an ordinal scale of 1–4, using the operational definitions of postoperative pulmonary complications described by Kroenke et al. [8] (Table 1) . Clinically significant respiratory complications were defined as one item among grade 3 or 4 complications.

**Table 1:** Operational definitions of postoperative pulmonary complications

Grade	Definition
1	Cough, dry Microatelectasis: abnormal lung findings and temperature > 37.5 °C without other documented cause; results of chest radiograph either normal or unavailable Dyspnea, not due to other documented cause
2	Cough, productive, not due to other documented cause Bronchospasm: new wheezing or pre-existent wheezing resulting in change of therapy Hypoxemia: alveolar–arterial gradient > 29 and symptoms of dyspnea or wheezing Atelectasis: radiological confirmation plus either temperature > 37.5 °C or abnormal lung findings Hypercarbia, transient, requiring treatment, such as naloxone or increased manual or mechanical ventilation as an adverse reaction to pulmonary medication
3	Pleural effusion, resulting in thoracentesis Pneumonia, suspected: radiological evidence without bacteriological confirmation Pneumonia, proved: radiological evidence and documentation of pathological organism by Gram stain or culture Pneumothorax Re-intubation postoperatively or intubation, period of ventilator dependence does not exceed 48 h
4	Ventilatory failure: postoperative ventilator dependence exceeding 48 h, or re-intubation with subsequent period of ventilator dependence exceeding 48 h

### Statistical analysis

Continuous variables are expressed as the mean ± standard deviation (SD) and were compared using Student’s *t* test or the Mann–Whitney test, as appropriate. Nominal variables are expressed as percentages and were analyzed using the *χ*^2^ test or Fisher’s exact probability test. All variables with *P* values of < 0.20 in the univariate analysis were included in the multivariable analyses. Other clinically relevant variables, namely, sex, age, body mass index (BMI), and BSA, were adjusted in the multivariable analysis. Independent predictors of postoperative complications after cardiac surgery were identified using a multivariate logistic regression model with the forced entry method. Odds ratios (OR), 95% confidence intervals (95%CI), and *P* values are reported. To minimize selection bias derived from the retrospective observational study design, propensity score analyses were performed to generate two groups, considering the following covariates: age, sex, BMI, %VC, and hemoglobin. 70 patients with %DL_CO_ < 70% and 67 patients with %DL_CO_ ≥ 70% were matched. A logistic regression analysis for the abovementioned covariates, with nearest-neighbor one-to-one matching, was performed to determine the propensity scores. All statistical tests were two-sided, and *P* values of < 0.05 were considered to indicate statistical significance. All statistical analyses were performed using the IBM SPSS statistics 24 software program (IBM, Armonk, NY, USA).

## Results

### Patient characteristics and outcomes

**Fig. 1:**
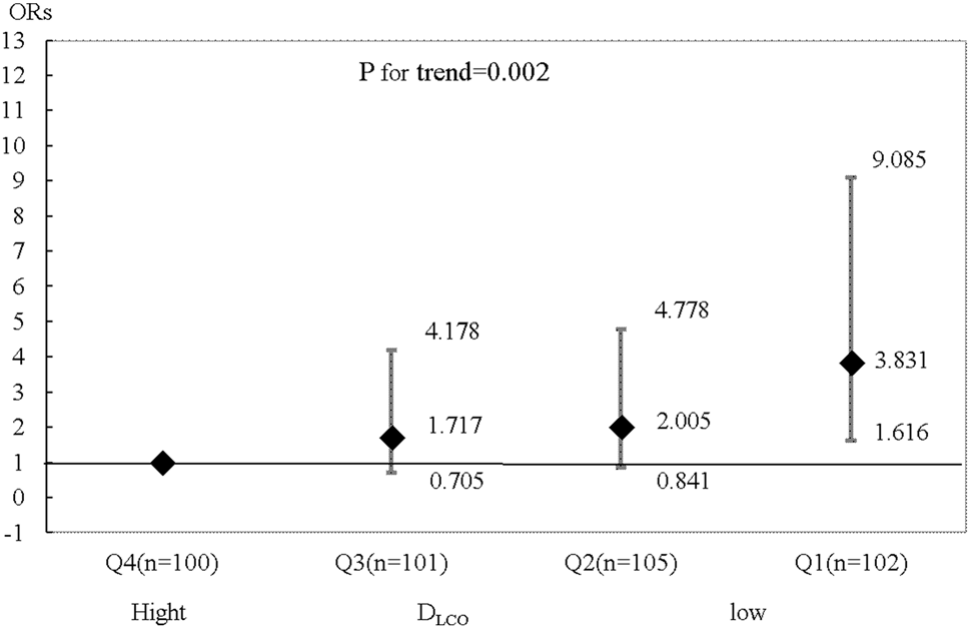
Patients with all complications after surgery and the %DL_CO_ quartiles. The %DL_CO_ quartiles were as follows: Q1 (≤ 74.6%), Q2 (74.7–88.8%), Q3 (88.9–101.7%), and Q4 (≥ 101.8%). *DL*_*CO*_ diffusing capacity of lung for carbon monoxide, *OR* odds ratio. Error bars represent 95% confidence intervals. OR adjusted for sex, age, body mass index, body surface area, hemoglobin A_1C_, New York Heart Association class, atrial fibrillation, brain natriuretic peptide, %vital capacity, hemoglobin, logistic Euro score II, STS score, durations of surgery, pump, and aortic clamp

**Fig. 2:**
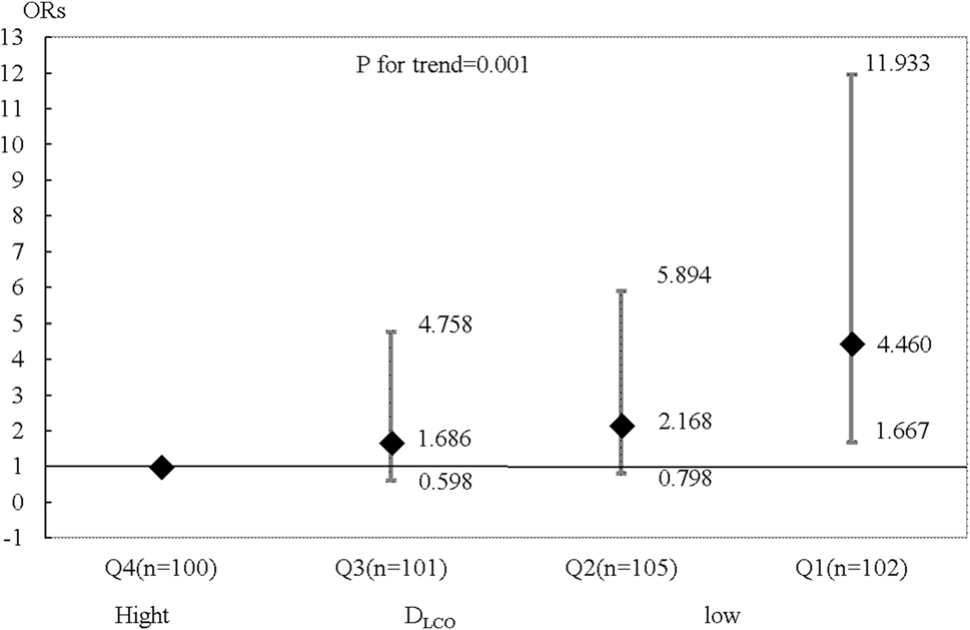
Patients with respiratory complications after surgery and the %DL_CO_ quartiles. The %DL_CO_ quartiles were as follows: Q1 (≤ 74.6%), Q2 (74.7–88.8%), Q3 (88.9–101.7%), and Q4 (≥ 101.8%). The ORs were adjusted as described in the Fig. 1 legend. *DL*_*CO*_ diffusing capacity of lung for carbon monoxide,* OR* odds ratio. Error bars represent 95% confidence intervals

Table 2 summarizes the characteristics of the 408 patients (age, 66.0 ± 10.0 years; male, *n* = 295 [72.3%]), whose data were analyzed in this study. Isolated coronary artery bypass grafting (CABG) was performed for 224 (54.9%) patients, and 184 (45.9%) underwent valve surgery (including concomitant cardiac surgery). Six (1.47%) patients died in hospital due to multi-organ failure (*n* = 1), sudden death (*n* = 1), and sepsis (*n* = 4). Operative complications developed in 91 (22.3%) patients and consisted of gastrointestinal disorder (*n* = 3), stroke (*n* = 4), renal disorder (*n* = 5), cardiac disorder (*n* = 7), wound infection (*n* = 19), and respiratory complications (*n* = 71; Grade 3: *n* = 61, Grade 4: *n* = 3). Figures 1 and 2 show the relationship between patients with all complications or respiratory complications and %DL_CO_ by quartile. The incidence of all complications significantly differed in Q1 (OR, 3.323; 95%CI, 1.472–7.500; *P* = 0.005); the OR for respiratory complications was 3.462 (95%CI, 1.434–8.357; *P* = 0.005). Although a DLco value of < 80% of the predicted value was considered abnormal, according to a previous definition by Steenhuis et al. [9], the incidence of complications differed in Q1 (%DLco < 74.6%). A DLco value of < 70% the predicted value was considered to be the cut-off value. The area under the receiver operating characteristic curve values was 0.625 (95%CI 0.558–0.692) for all complications and 0.632 (95%CI 0.557–0.707) for respiratory complications. The sensitivity and specificity of %DLco, with a cut-off value of 70%, were 0.864 and 0.297, respectively, for all complications (0.861 and 0.324 for respiratory complications).

**Table 2:** Demographics and clinical variables stratified by %DL_CO_ risk

	All patients (*n* = 408)	%DL_CO_ ≥ 70% (*n* = 338)	%DL_CO_ < 70% (*n* = 70)	*P*
Female, *n* (%)	113 (27.7)	86 (25.4)	27 (38.6)	0.025
Age (years)	66 ± 10.0	66.5 ± 9.7	63.8 ± 11.2	0.036
BMI (kg/m^2^)	23.6 ± 3.5	23.6 ± 3.5	23.3 ± 3.4	0.412
BSA (m^2^)	1.64 ± 0.19	1.64 ± 0.19	1.62 ± 0.19	0.413
Hypertension, *n* (%)	330 (80.9)	276 (81.7)	54 (77.1)	0.382
Hyperlipidemia, *n* (%)	264 (64.7)	219 (64.8)	45 (64.3)	0.936
Smoking, *n* (%)	264 (64.7)	213 (63.0)	51 (72.9)	0.117
Hemoglobin A_1C_, (%)	6.0 (1.0)	6.0 (0.9)	6.2 (1.3)	0.113
NYHA class (I, II), *n* (%)	332 (81.4)	275 (81.4)	57 (81.4)	0.382
Recent AMI, *n* (%)	17 (4.2)	14 (4.1)	3 (4.3)	0.956
Atrial fibrillation, *n* (%)	79 (19.4)	64 (18.9)	15 (21.4)	0.631
Ex. arteriopathy, *n* (%)	73 (17.9)	52 (15.4)	21 (30.0)	0.004
EF (%)	57.4 ± 13.7	57.7 ± 13.7	56.0 ± 14.0	0.345
BNP (pg/mL)	205.93 ± 403.7	193.6 ± 381.3	265.3 ± 497.1	0.023^*^
%VC (%)	94.6 ± 16.3	95.6 ± 16.1	89.6 ± 16.5	0.005
FEV_1.0_% (%)	72.6 ± 10.6	72.6 ± 10.1	72.4 ± 12.6	0.878
PaO_2_ (mmHg)	89.1 ± 13.2	89.2 ± 2.9	88.5 ± 14.8	0.690
PaCO_2_ (mmHg)	39.7 ± 3.9	39.8 ± 3.9	39.2 ± 4.3	0.328
Hemoglobin (g/dL)	13.0 ± 1.8	13.1 ± 1.7	12.3 ± 1.9	< 0.001
Creatinine (mg/dL)	1.3 ± 1.7	1.3 ± 1.7	1.3 ± 1.7	0.306^*^
Euro score II	2.05 ± 1.80	1.99 ± 1.75	2.34 ± 1.99	0.051^*^
STS score	2.25 ± 2.77	2.21 ± 2.73	2.48 ± 2.98	0.516^*^
CABG only, *n* (%)	224 (54.9)	193 (57.1)	31 (44.3)	0.050
Operative time (min)	324.2 ± 80.2	322.5 ± 79.2	332.3 ± 85.3	0.350
Pump time (min)	145.7 ± 50.2	144.3 ± 49.9	152.0 ± 51.5	0.244
Aortic clamp time (min)	105.97 ± 42.3	105.0 ± 42.6	110.2 ± 40.5	0.347
All complications, *n* (%)	91 (22.3)	64 (18.9)	27 (38.6)	< 0.001
Resp. complication, *n* (%)	71 (17.4)	48 (14.2)	23 (32.9)	< 0.001
Hospital mortality, *n* (%)	6 (1.5)	4 (1.2)	2 (2.9)	0.275

Continuous data are presented as mean ± SD

*BMI* body mass index, *BNP* brain natriuretic peptide, *BSA* body surface area, *CABG* coronary artery bypass graft, *EF* ejection fraction, *Ex*. arteriopathy extracardiac arteriopathy, *FEV*_*1.0*_*%* percent predicted forced expiratory volume in 1 s, *NYHA* New York Heart Association, *%VC* percent predicted vital capacity, Recent *AMI* acute myocardial infarction within 3 months, *Resp* respiratory, *ST,S* Society of Thoracic Surgeons, *%DL*_*CO*_ percent predicted diffusing capacity of lung for carbon monoxide

*Fisher exact test or Mann–Whitney test

### Preoperative demographics and clinical variables according to the %DL_CO_ risk

Table 2 shows the preoperative and perioperative factors of patients with %DL_CO_ of ≥ 70% (*n* = 338) or < 70% (*n* = 70). Significant differences were observed in age (66.5 ± 9.70 vs. 63.8 ± 11.2 years; *P* = 0.036) and sex (female) (86 [25.4%] vs. 27 [38.6%]; *P* = 0.025). There were no significant differences in the risk factors, which included hypertension, hyperlipidemia, history of smoking, BMI, BSA, and HbA_1C_. Among the clinical and biochemical parameters, significant differences were observed in extracardiac arteriopathy (52 [15.4%] vs. 21 [30.0%]; *P* = 0.004) and Hb (13.1 ± 1.7 vs. 12.3 ± 1.9 g/dL; *P* < 0.001). In terms of the cardiac function, the brain natriuretic peptide (BNP) levels of the two groups were significantly different (193.6 ± 381.3 vs. 265.3 ± 497.1 pg/mL; *P* = 0.023), whereas ejection fraction was not (57.7% ± 13.7% vs. 56.0% ± 14.0%; *P* = 0.345). Among the factors associated with the respiratory function, %VC was significantly different (95.65 ± 16.1% vs. 89.6 ± 16.5%; *P* = 0.005), whereas FEV_1.0_% was not (72.6 ± 10.1% vs. 72.4 ± 12.6%; *P* = 0.878). Among the factors associated with the renal function, the serum creatinine level did not differ to a statistically significant extent (1.3 ± 1.7 vs. 1.3 ± 1.7 mg/dL; *P* = 0.306).

CABG was the only operative method for which there was significant difference (193 [57.1%] vs. 31 [44.3%] *P* = 0.050). The operative time (322.5 ± 79.2 vs. 323 ± 85.3 min; *P* = 0.350), pump time (144.3 ± 49.9 vs. 152.0 ± 51.5 min; *P* = 0.244), and aortic clamp time (105.0 ± 22.6 vs. 110.2 ± 40.5 min; *P* = 0.347) did not differ to a statistically significant extent. Furthermore, there was no significant difference in the rate of hospital mortality (4 [1.2%]) vs. 2 [2.9%]; *P* = 0.275. There were significant differences between the two groups in the rates of all complications (64 [18.9%] vs. 27 [38.6%]; *P* < 0.001) and respiratory complications (48 [14.2%] vs. 23 [32.9%]; *P* < 0.001).

### %DL_CO_ as a predictor of complications after cardiac surgery

Table 3 shows the results of the univariate analysis of patients with all complications and those with respiratory complications. Among the preoperative data, significant differences were observed in the rates of %DL_CO_ < 70%, BNP ≥ 100 pg/mL, Hb < 11 g/dL, and the Euro Score II and STS score values of the patients with and without all and those with and without respiratory complications. Among the perioperative factors, significant differences were observed in the operative time, the pump time and the aortic clamp time between the patients with and without complications. A multivariate logistic regression analysis identified BMI (OR, 1.156; 95%CI, 1.039–1.286; *P* = 0.008), BSA (OR, 0.040; 95%CI, 0.003–0.575; *P* = 0.018), and a reduced %DL_CO_ (OR, 2.682; 95%CI, 1.449–4.962; *P* = 0.002) as preoperative factors that were significant independent predictors of all complications. Pump time (OR, 1.016; 95%CI, 1.003–1.030; *P* = 0.017) as identified as a perioperative factor that was a significant predictor of all complications (Table 4). The multivariate logistic regression analysis identified a reduced %DL_CO_ (OR, 2.833; 95%CI, 1.490–5.398; *P* = 0.001) and increased HbA_1C_ (OR, 2.284; 95%CI. 1.102–4.733; *P* = 0.026) as preoperative factors that were significant independent predictors of respiratory complications (Table 5). The propensity score analysis identified a reduced %DL_CO_ as a predictor of all complications and respiratory complications: all complications (OR, 3.270; 95%CI, 1.356–7.882; *P* = 0.008) and respiratory complications (OR, 3.447; 95%CI, 1.343–8.846; *P* = 0.010) (Table 6).

**Table 3:** Demographics of patients and the clinical variables according to complications

	All complications		*P*	Respiratory complications		*P*
	Absent (*n* = 317)	Present (*n* = 91)		Absent (*n* = 337)	Present (*n* = 71)	
Sex, female, *n* (%)	85 (26.8)	28 (30.8)	0.457	89 (26.4)	24 (33.8)	0.206
Age ≥ 75 years, *n* (%)	62 (19.6)	20 (22.0)	0.612	65 (19.3)	17 (23.9)	0.374
BMI (kg/m^2^)	23.6 ± 3.4	23.6 ± 3.8	0.912	23.6 ± 3.4	23.4 ± 3.8	0.610
BSA (m^2^)	1.64 ± 0.2	1.61 ± 0.09	0.147	1.64 ± 0.19	1.6 ± 0.19	0.069
Hypertension, *n* (%)	255 (80.4)	75 (82.4)	0.673	272 (80.7)	58 (81.7)	0.849
Hyperlipidemia, *n* (%)	208 (65.6)	56 (61.5)	0.473	221 (65.6)	43 (60.6)	0.442
Smoking, *n* (%)	202 (63.7)	62 (68.1)	0.438	219 (65.0)	45 (63.4)	0.797
Hemoglobin A1c ≥ 7%, *n* (%)	45 (14.4)	19 (21.1)	0.123	47 (14.2)	17 (23.9)	0.041
NYHA grade > III, *n* (%)	54 (17.0)	22 (24.2)	0.123	58 (17.2)	18 (25.4)	0.109
Recent AMI, *n* (%)	15 (4.7)	2 (2.2)	0.286	15 (4.5)	2 (2.8)	0.531
Atrial fibrillation, *n* (%)	56 (17.7)	23 (25.3)	0.105	60 (17.8)	19 (26.8)	0.083
Ex. arteriopathy, *n* (%)	57 (18.0)	16 (17.6)	0.930	60 (17.8)	13 (18.3)	0.920
EF < 40%, *n* (%)	35 (11.0)	13 (14.3)	0.397	38 (11.3)	10 (14.1)	0.504
BNP ≥ 100 pg/mL, *n* (%)	140 (44.7)	54 (60.0)	0.011	152 (45.8)	42 (59.2)	0.041
%DL_CO_ < 70%, *n* (%)	43 (13.6)	27 (29.7)	< 0.001	47 (13.9)	23 (32.4)	< 0.001
%VC < 80%, *n* (%)	45 (14.2)	20 (22.0)	0.074	50 (14.8)	15 (21.1)	0.188
FEV_1.0_% < 75%, *n* (%)	158 (49.8)	43 (47.3)	0.663	167 (49.6)	34 (47.9)	0.798
PaO_2_ < 80 mmHg, *n* (%)	69 (23.3)	16 (18.8)	0.381	72 (22.9)	13 (19.7)	0.575
PaCO_2_ ≥ 40 mmHg, *n* (%)	147 (49.7)	39 (45.9)	0.539	157 (49.8)	29 (43.9)	0.383
Hemoglobin, < 11 g/dL, *n* (%)	36 (11.4)	19 (20.9)	0.019	40 (11.9)	15 (21.1)	0.038
Creatinine ≥ 2.0 mg/dL, *n* (%)	23 (7.3)	9 (9.9)	0.410	26 (7.7)	6 (8.5)	0.834
Euro Score II	1.90 ± 1.58	2.59 ± 2.34	0.001^*^	1.91 ± 1.57	2.72 ± 2.53	0.003^*^
STS score	2.04 ± 2.51	3.01 ± 3.44	0.002^*^	2.09 ± 2.57	3.06 ± 3.49	0.006^*^
CABG alone, *n* (%)	178 (56.2)	46 (50.5)	0.344	188 (55.8)	36 (50.7)	0.434
Operative time (min)	318.7 ± 79.8	343.1 ± 79.4	0.010	320.5 ± 79.6	341.5 ± 81.6	0.045
Pump time (min)	140.7 ± 45.2	163 ± 61.9	0.002	142.3 ± 45.7	161.7 ± 65.9	0.020
Aortic clamp time (min)	103 ± 38.8	116.2 ± 51.4	0.025	104 ± 39.4	115.2 ± 53.1	0.094

Continuous data are presented as mean ± SD

*BMI* body mass index, *BNP* brain natriuretic peptide, *BSA* body surface area, *CABG* coronary artery bypass graft, *EF* ejection fraction, *Ex*. arteriopathy extracardiac arteriopathy, *FEV*_*1.0*_*%* percent predicted forced expiratory volume in 1 s, *NYHA* New York Heart Association, *%VC* percent predicted vital capacity, Recent *AMI* acute myocardial infarction within 3 months, *Resp* respiratory, *STS* Society of Thoracic Surgeons, *%DL*_*CO*_ percent predicted diffusing capacity of lung for carbon monoxide

*Fisher exact test or Mann–Whitney test

**Table 4:** Univariate and multivariate analyses of the predictors of all complications

	Univariate analysis			Multivariate analysis		
	OR	(95%CI)	*P*	OR	(95%CI)	*P*
Sex (female)	1.213	(0.729–2.020)	0.458	0.463	(0.199–1.076)	0.074
Age (≥ 75 years)	1.159	(0.656–2.046)	0.612	0.745	(0.354–1.566)	0.438
BMI (kg/m^2^)	1.004	(0.939–1.073)	0.917	1.156	(1.039–1.286)	0.008
BSA (m^2^)	1.034	(0.933–1.147)	0.148	0.040	(0.003–0.575)	0.018
Hemoglobin A1c (≥ 7%)	1.539	(0.859–2.758)	0.147	1.744	(0.872–3.4862)	0.116
NYHA class (III, IV)	0.594	(0.878–2.743)	0.126	1.520	(0.794–2.909)	0.206
Atrial fibrillation	1.576	(0.906–2.743)	0.107	1.338	(0.660–2.714)	0.420
BNP (≥ 100 pg/mL)	1.854	(1.150–2.986)	0.011	1.091	(0.614–1.940)	0.767
%DL_CO_ (< 70%)	2.688	(1.547–4.673)	< 0.001	2.682	(1.449–4.962)	0.002
%VC (< 80%)	1.703	(0.946–3.065)	0.076	1.243	(0.620–2.491)	0.540
Hemoglobin (< 11 g/dL)	2.060	(1.116–3.803)	0.021	1.306	(0.623–2.735)	0.479
Euro score II	1.204	(1.069–1.056)	0.002	1.028	(0.857–1.233)	0.763
STS score	1.113	(1.033–1.200)	0.005	1.080	(0.964–1.211)	0.185
Operative time (min)	1.004	(1.001–1.006)	0.012	0.999	(0.995–1.004)	0.752
Pump time (min)	1.008	(1.004–1.013)	< 0.001	1.016	(1.003–1.030)	0.017
Aortic clamp time (min)	1.007	(1.002–1.012)	0.01	0.988	(0.975–1.002)	0.085

*OR* odds ratio, *CI* confidence interval, *BMI* body mass index, *BNP* brain natriuretic peptide, *BSA* body surface area, *NYHA* New York Heart Association, *%VC* percent predicted vital capacity, *STS* Society of Thoracic Surgeons, *%DL*_*CO*_ percent predicted diffusing capacity of lung for carbon monoxide

**Table 5:** Univariate and multivariate analyses of predictors of respiratory complications

	Univariate analysis			Multivariate analysis		
	OR	(95%CI)	*P*	OR	(95%CI)	*P*
Sex (female)	1.423	(0.822–2.462)	0.207	0.587	(0.239–1.440)	0.245
Age (≥ 75 years)	1.317	(0.717–2.421)	0.375	0.942	(0.429–2.068)	0.882
BMI (kg/m^2^)	1.002	(0.591–1.77)	0.941	1.099	(0.979–1.233)	0.108
BSA (m^2^)	0.278	(0.070–1.109)	0.070	0.062	(0.003–1.096)	0.058
HbA1c (≥ 7%)	1.910	(1.020–3.571)	0.043	2.284	(1.102–4.733)	0.026
NYHA class (III, IV)	1.634	(0.892–2.991)	0.112	1.512	(0.751–3.261)	0.246
Atrial fibrillation	1.687	(0.930–3.058)	0.085	1.516	(0.705–3.261)	0.287
BNP (≥ 100 pg/mL)	1.720	(1.020–2.885)	0.042	0.962	(0.514–1.802)	0.904
%DL_CO_ (< 70%)	2.960	(1.647–5.306)	< 0.001	2.833	(1.490–5.388)	0.001
%VC (< 80%)	1.537	(0.807–2.928)	0.191	1.022	(0.475–2.196)	0.956
Hemoglobin (< 11 g/dL)	1.989	(1.029–3.842)	0.041	1.356	(0.621–2.963)	0.445
Euro score II	1.226	(1.084–1.088)	0.001	1.059	(0.876–1.281)	0.553
STS score	1.106	(1.024–1.195)	0.011	1.044	(0.922–1.182)	0.498
Operative time (min)	1.003	(1.000–1.006)	0.047	0.999	(0.994–1.004)	0.703
Pump time (min)	1.007	(1.002–1.032)	0.004	1.013	(0.999–1.028)	0.066
Aortic clamp time (min)	1.006	(1.000–1.012)	0.043	0.990	(0.976–1.004)	0.168

*OR* odds ratio, *CI* confidence interval, *BMI* body mass index, *BNP* brain natriuretic peptide, *BSA* body surface area, *NYHA* New York Heart Association, *%VC* percent predicted vital capacity, *STS* Society of Thoracic Surgeons, *%DL*_*CO*_ percent predicted diffusing capacity of lung for carbon monoxide

**Table 6:** Propensity score analyses of predictors of all and respiratory complications

	All complications			Respiratory complications		
	OR	(95% CI)	*P*	OR	(95% CI)	*P*
BSA (m^2^)	0.446	(0.040–4.943)	0.511	0.273	(0.021–3.504)	0.319
HbA1c (≥ 7%)	0.577	(0.149–2.241)	0.427	0.959	(0.245–3.763)	0.953
NYHA class (III,IV)	2.416	(0.797–7.322)	0.119	1.965	(0.616–6.268)	0.254
Atrial fibrillation	1.997	(0.650–6.136)	0.227	1.547	(0.486–4.923)	0.460
BNP (≥ 100 pg/mL)	0.760	(0.296–1.954)	0.569	0.818	(0.302–2.213)	0.692
%DL_CO_ (< 70%)	3.270	(1.356–7.882)	0.008	3.447	(1.343–8.846)	0.010
Euro score II	1.068	(0.783–1.455)	0.678	1.174	(0.853–1.615)	0.325
STS score	1.050	(0.882–1.249)	0.586	0.968	(0.800–1.172)	0.740
*Operative time* (min)	1.005	(0.997–1.013)	0.212	1.003	(0.995–1.012)	0.424
Pump *time* (min)	1.002	(0.981–1.024)	0.828	1.003	(0.980–1.026)	0.818
Aorta clamp *time* (min)	1.001	(0.978–1.024)	0.945	1.004	(0.981–1.029)	0.711

Propensity scores were calculated by age, sex, BMI, %VC and hemoglobin, and 70 patients with %DL_CO_ < 70% and 67 patients with %DL_CO_ ≥ 70% were matched

*OR* odds ratio, *CI* confidence interval, *BMI* body mass index, *BSA* body surface area, *NYHA* New York Heart Association, *%VC* percent predicted vital capacity, *STS* Society of Thoracic Surgeons, *%DL*_*CO*_ percent predicted diffusing capacity of lung for carbon monoxide

## Discussion

The principal finding of this study was that the preoperative DL_CO_ was correlated with postoperative complications after cardiac surgery. Others have described significant and prolonged impairment of the pulmonary function after cardiac surgery [1]. Decreased ventilation, pulmonary disease, and reduced alveolar perfusion caused by poor cardiac output and chronic heart failure might also influence DL_CO_ [10]. DL_CO_ is a clinically useful indicator of the lung function, because it assesses gas transfer from the alveoli to the red blood cells. The preoperative DL_CO_ is not routinely measured in patients in most cardiac surgery units. Reduced postoperative capillary filtration due to basal membrane thickening, enhanced alveolar fluid clearance, and increased lymphatic drainage leads to restricted lung spirometry and impaired gas transfer [6]. We hypothesized that the postoperative DL_CO_ might be more decreased than the preoperative DL_CO_ and that this could serve as a predictor of early complications after cardiac surgery. The present study found that more postoperative complications developed among patients with %DL_CO_ of < 70% than among those with %DL_CO_ of > 70%. A previous study also found that patients with stable chronic heart failure had decreased %VC values, in addition to decreased DL_CO_ and *D*_M_ values [11]. The present study showed that the %VC values were decreased and the BNP levels were increased in patients with lower DL_CO_ values; however, these patients might have had preoperative chronic heart failure. Thus, %DL_CO_ might be a marker of heart failure.

A previous study suggested that cardiac surgery may also contribute to a greater reduction in DL_CO_. The mechanism underlying the reduction of DL_CO_ after cardiac surgery is unclear. One hypothesis is that it might reflect pathophysiological changes in the pulmonary microcirculation initiated by CPB, such as a systemic inflammatory response with coagulopathy and altered microvascular permeability [12]. That CPB interferes with pulmonary function has been established. It can induce adverse effects on alveolar stability by activating the complement cascade, sequestering neutrophils in the pulmonary microvascular bed, releasing oxygen-derived free radicals, and changing the composition of alveolar surfactant [13]. The mechanism underlying the diffusion impairment after cardiac surgery could be caused by pulmonary edema and the accumulation of fluid in interstitial spaces, ventilation–perfusion mismatches, or changes in Hb concentrations [14]. A few studies have identified a relationship between DL_CO_ and the outcomes after cardiac surgery. Published data show that a %DL_CO_ value of < 50% the predicted value at the preoperative PFT is an independent risk factor for a > threefold increase in mortality after adjustment for mortality risk estimates [15]. Few patients in the present study had a %DL_CO_ value of < 50%. Thus, our analysis included %DL_CO_ < 70% as an approximation for Q1. The findings of the present study showed that %DL_CO_ < 70% in a preoperative PFT was independently associated with a > 3.3-fold increase in risk for all complications after adjustment for morbidity risk estimates; the risk of respiratory complications was increased > 3.4-fold.

Postoperative respiratory complications continue to affect patient morbidity and mortality, length of hospital stay, and overall resource utilization, despite advances in preoperative, intraoperative. and postoperative care [16–18]. Respiratory muscle dysfunction due to surgery can lead to a reduced vital capacity, tidal volume, and total lung capacity [19]. This could cause atelectasis in the basal lung segments and decrease the functional residual capacity, which affects pulmonary gas exchange properties by increasing ventilation/perfusion mismatches. Thus, DL_CO_ might also decrease after surgery. Preoperative and postoperative chest physical therapy has significantly reduced the number of patients who develop atelectasis, but it does not significantly benefit patients who develop respiratory complications due to infection [20]. Improving the preoperative respiratory status of these patients via the fine adjustment of medication therapy and strict physiotherapeutic control seems important. Preoperative short-term pulmonary rehabilitation for such patients improves the pulmonary function and reduces the incidence of atelectasis, consolidation, and pneumothorax [16]. Preoperative physical therapy with inspiratory muscle training for at least 2 weeks reduced the incidence of postoperative pulmonary complications by 50% [18]. Although the present study did not uncover evidence as to whether surgical outcomes would improve with preoperative short-term pulmonary rehabilitation, determining the correct timing of surgery is also important for avoiding respiratory decompensation.

The present study is associated with several limitations. Although all data were prospectively recorded, this was a retrospective, single-institute study. The retrospective design is susceptible to various sources of bias, which might have not been identified or controlled. The preoperative PFTs were performed according to requests from clinicians, who were not blinded to the results of the PFT. Thus, the possibility that patient management might have been affected by the PFT results cannot be excluded.

In conclusion, the %DL_CO_ seems to be a powerful predictor of postoperative complications. To the best of our knowledge, this is one of the few studies to assess whether DL_CO_ is a potential risk factor for adverse outcomes of patients after cardiac surgery. Preoperative DL_CO_ values might provide more accurate prognostic information about outcomes after cardiac surgery. Preoperative PFT findings might provide clinicians with more accurate risk profiles as well as additional prognostic information. Thus, pulmonary function testing, including measurement of DL_CO_, should be a routine component of preoperative evaluations.
